# Controversies in acute multiligamentary knee injuries (MLKI)

**DOI:** 10.1186/s40634-020-00260-8

**Published:** 2020-07-27

**Authors:** Manuel F. Mosquera, Alejandro Jaramillo, Ricardo Gil, Yessica Gonzalez

**Affiliations:** 1 Clinica Erasmo, Valledupar, Colombia; 2 Clinica La Carolina, Carrera 14 # 127-11 Cons 307-308, Bogota, Colombia; 3 Clinica Del Norte, Medellin, Colombia

**Keywords:** Multi-ligamentary knee injuries, Knee dislocation, Multi-ligament knee surgery

## Abstract

**Summary:**

Multiligament injuries of the knee (MLKI), remain an infrequent pathology especially in developed countries compared to mono-ligament lesions. In Colombia, MLKI is frequent due to the high accident rate on motorcycles. In the city of Bogota alone, about 160 motorcycle accidents have been estimated daily, being one of the cities that proportionately use this means of transport less compared to small cities. The term MLKI, include all ruptures of two or more major ligaments and therefore it has a broad spectrum of clinical presentation which creates a great challenge for the orthopedists and the surgeons envolved in this topic. The literature is rich in studies level IV but very poor in level I and level II, which generates controversies and little consensus in the diagnosis and treatment of this pathology. However there has been a gradual and better understanding of all factors involved in the treatment of MLKI that has improved the functional results of these knees in our patients, in fact we currently are more precise to achieve accurate diagnosis, evolved from not surgical approach to operate most, applying new anatomical and biomechanical concepts, with specialized and skill surgical techniques with more stable and biocompatible fixation implants, which allow in most cases to initiate an early integral rehabilitation program. Nevertheless due to the complexity and severity of the lesions, in some patients the functional results are poor. The goal of this revision is to identify the most frequent controversies in the diagnosis and treatment of MLKI, defining which of them are agreed according to what is reported in the literature and share some concepts based from the experience of more than 25 years of the senior author (MM) in the management of these injuries.

**Level of Evidence:**

V – Expert Opinion.

## Introduction

Multi-ligament knee injuries (MLKI) have been defined as those that are accompanied by rupture of two or more of the four major ligaments, referring to the cruciate ligaments and collaterals, although some identify six [63]. Schenck et al. [96, 97, 117] classified the knee dislocations in five degrees according to the injured ligaments and not by the direction of displacement as it was traditionally done, because most of the dislocations came reduced into the emergency room and were unclassificable [118]*.* The number of ligaments involved, which of them are injured and the associated injuries is closely related to the magnitude of the trauma and the mechanism of production of the lesion [34]. In general, high-energy trauma usually injure three or the four major ligaments and those of low energy up to two ligaments, usually one cruciate and one collateral [102], but in some special conditions such as morbid obesity, ultra-low-energy trauma [5, 33] causes severe injuries that it can include the four major ligaments. Although the MLKI is of rare presentation in many countries [3, 11, 25, 59] in Colombia is a frequent pathology in the emergency services due to the high rate of accidents that occur in motorcycles, especially in small cities.

**Fig. 1:**
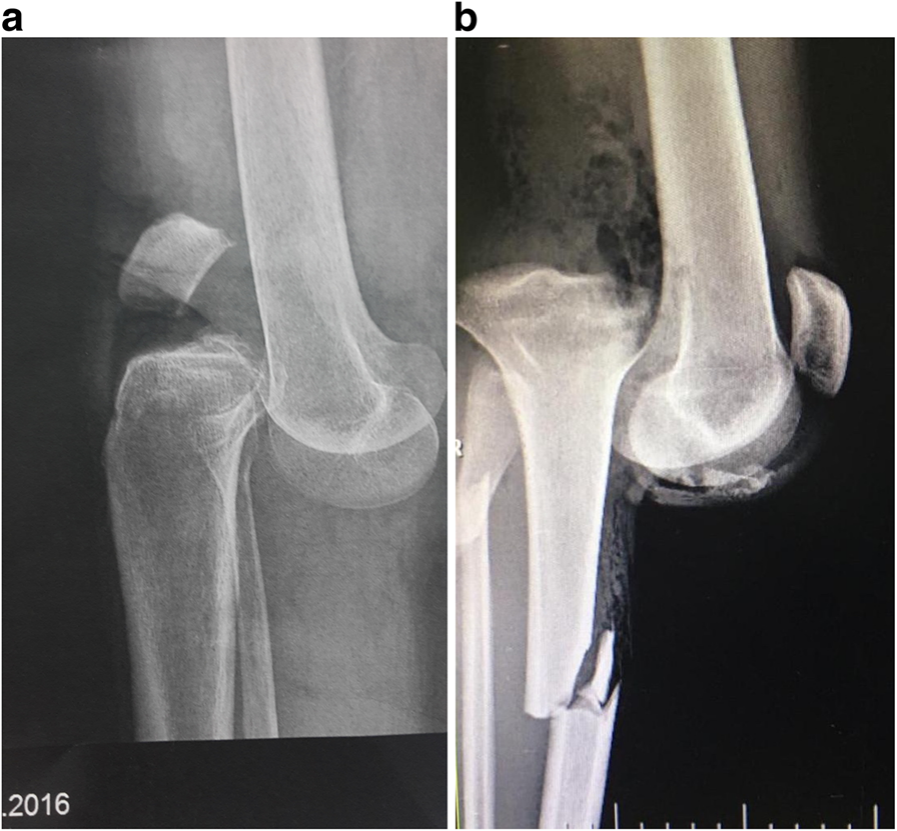
**a** knee dislocation KDIV. **b** Knee dislocation KDV

The majority of MLKI are product of a knee dislocation, (Fig. 1a knee dislocation KDIV), (Fig. 1b, Knee dislocation KDV) and the importance of suspect and diagnose it, is related by the high degree of association of lesion of the popliteal artery, which varies according to the series between 10 to 65% [16, 37, 73, 110] . Even when we diagnose a knee dislocation, a neuro-vascular lesion may go unnoticed if the patient is not properly examined and if some simple tests such as the Ankle-Brachial index that can alert us to a popliteal arterial lesion are not used [25, 73, 119] On some occasions when patients arrive unconscious in the emergency department, a peroneal nerve lesion may be neglected which can have serious consequences [35, 58, 82].

## Controversies in diagnosis and treatment

Given the hetereogenicity of multi-ligament injuries of the knee, its broad spectrum of clinical presentation and the poor level I-II evidence reported in the literature regarding all aspects of treatment, there are many controversies that have not been agreed upon by the experts [19], for this reason, this review intends to give to the reader some treatment guidelines in such controversial aspects, supported by the evidence that exist and for the authors experience mainly by the senior (MM), who has dedicated more than 25 years of his professional career in dealing with this pathology.

To start with, the only “consensus” that currently exists in the treatment of MLKI in Schenck KDII / III / IV lesions, is that operated patients have a better outcome than those treated without surgery [20, 52, 54, 90, 93] in terms of stability, work return and quality of life. Although there are reports of patients operated by MLKI who have returned to their same sporting level [56, 104] it is usual for their level of return to work and sport to be very low [24, 40] therefore these injuries can never be compared with isolated lesions of the cruciates or grade KDI of the Schenck classification in terms of their clinical results. It is not clear in the literature what it means have a “good result” in multi-ligament surgery, however it is generally accepted that the goals are to achieve a “stable” and functional joint, even though a considerable number of patients in their evaluations and arthrometric test, have residual laxity which is often not symptomatic.

**Fig. 2:**
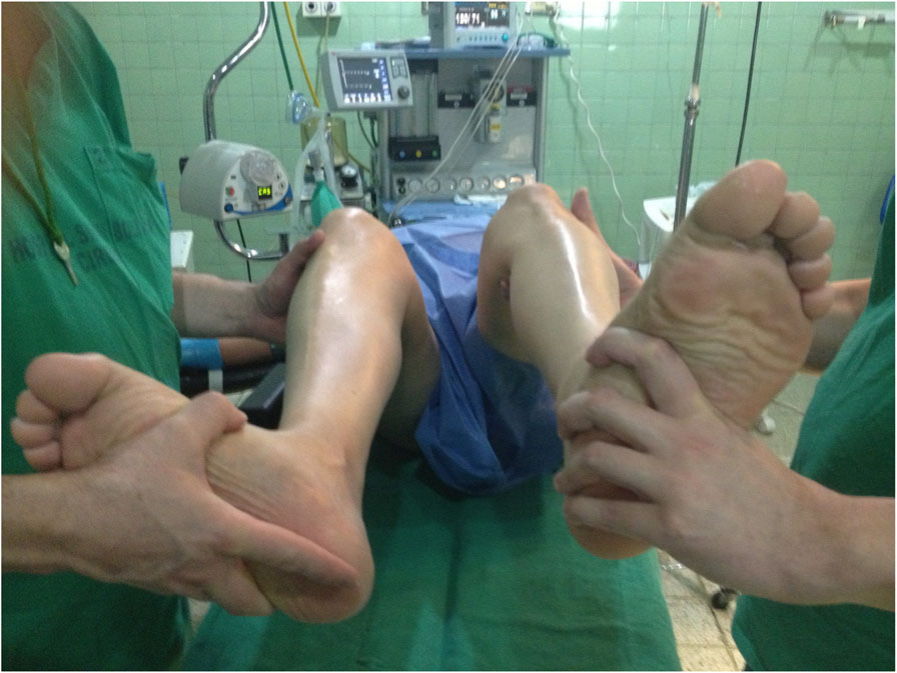
Dial Test at 30°

Except in unconscious patients, in the authors article experience, the clinical examination of the knee to establish which ligaments are compromised, is painful which obliges the surgeon to diagnose injuries by inspection and palpation of the knee and for the use of tests that mobilize the knee in short arcs of flexion, such as the traditional and inverted lachman test, the recurvatum and external rotation test, the dial test, (Fig. 2 Dial Test at 30°) and the lateral and medial opening in flexion and extension. With these tests and with the help of magnetic resonance imaging (MRI), 100 % certainty of which major ligaments are compromised can be reached.

The **First controversy** that arises is to know what the ideal test is if there is a high suspicion of vascular injury after having performed the ankle-brachial index [67]. There is no agreement in the literature if the arteriography is superior to angio-tac (CTA) or angio-resonance, but taking into account the sensitivity, specificity, non-invasiveness, lower morbidity and the better availability of CTA in the emergency department, it is preferred, to make a surgical decision of exploration and revascularization [10, 88]. In these cases, the use of an external fixator after the procedure is mandatory and non-controversial [72]. In the authors article opinion, given that the vascular lesion in the multi-ligament injuries that come from a knee dislocation are high [74], even with a normal pedal pulse, it is mandatory in all patients to perform the ankle-brachial index to rule out injuries that due to their characteristics may go unnoticed with catastrophic consequences. Therefore, Mills et al. [76] in a prospective study found that in all patients in whom the index was below 0.90, there was an arterial lesion that required repair / grafting. Anyway, if the index is not conclusive or is doubtful, is mandatory to make a CTA or Arteriography. We have keep in mind that certain vessels lesions, tear only the intimate layer making the diagnosis even more difficult and reconstruct this knees could have catastrophic consequences.

**Fig. 3:**
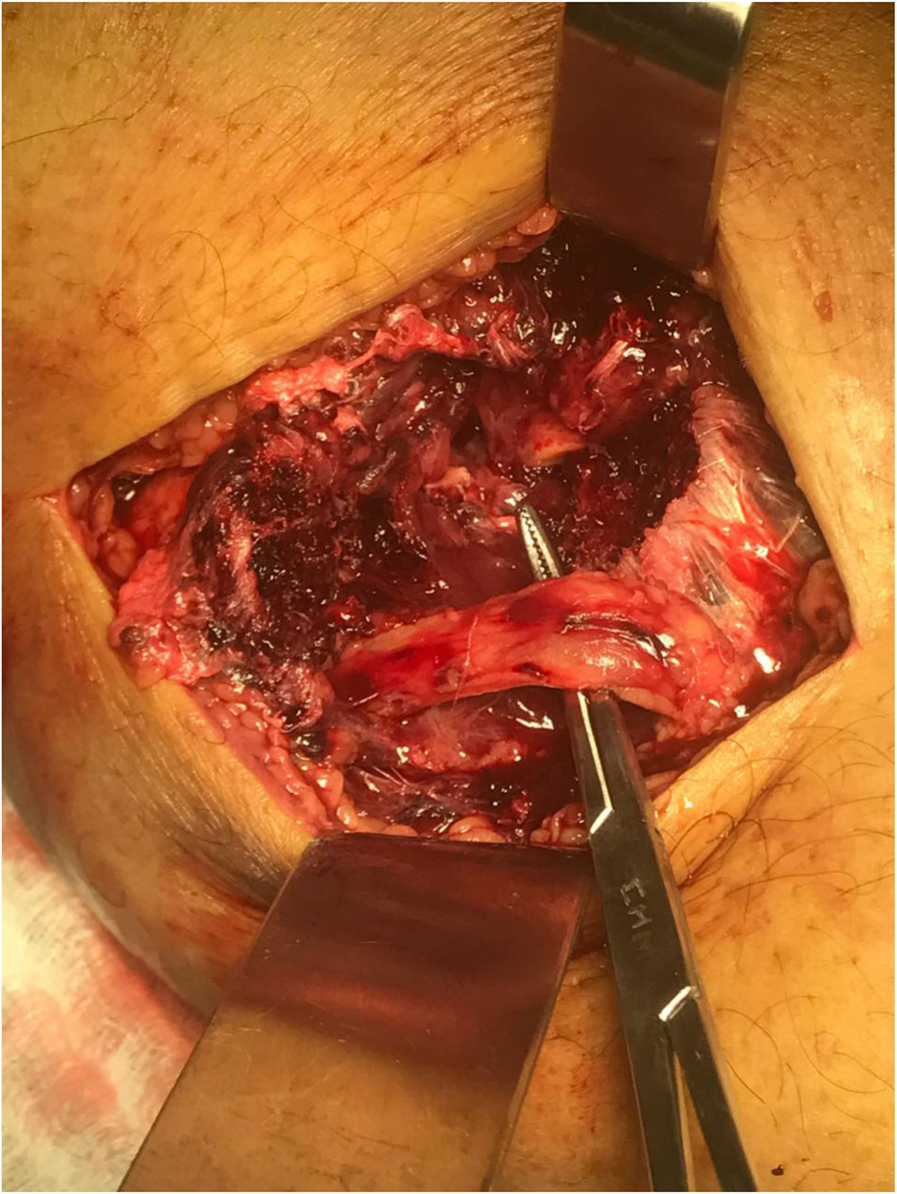
Peroneal Contusion. Courtesy of Ricardo Garcia MD

The **Second controversy** refers to the appropriate treatment of the peroneal nerve lesions, which can occur between 5% and 40% in a knee dislocation [42, 82] where in some special conditions such as in the obese patients, the risk may be greater [89]. There is no level I-II evidence, if acute exploration is necessary or conversely, the evolution should be follow up with clinical and electrophysiology control. Wodmass et al. [122] in a systematic review reported that only 40% of patients who have complete lesions recover active ankle dorsiflexion and 87% when the lesions are partial. In an article published by Samson et al. [95], they recommend in their treatment algorithm in continuous nerve lesions, early neurolysis with weekly and electrophysiological clinical control, and re-exploration if there is no progression in the improvement of symptoms and electromyographic findings. In complete lesions without tissue loss, these should be repaired early and augmented with grafts usually taken from the Sural Nerve, but in severe lesions with tissue loss the best option that is showing better results are nerve transfers [36, 121] Garozzo et al. [30, 31] and Ferrasi [27] have reported excellent results of peroneal nerve injuries performing early exploration, repair and nerve grafting, as well as transfer of the posterior tibial tendon. In the authors article opinion, clinical injury of the peroneal nerve is one of the indications for early surgery, ideally before two weeks and this should always be accompanied by the hand of a surgeon expert in peripheral nerve injuries (Fig. 3. Peroneal Contusion. Courtesy of Ricardo Garcia MD). The delay in the treatment of these lesions generate poor functional results even in knees that have achieved good joint stability [123].

**Fig. 4:**
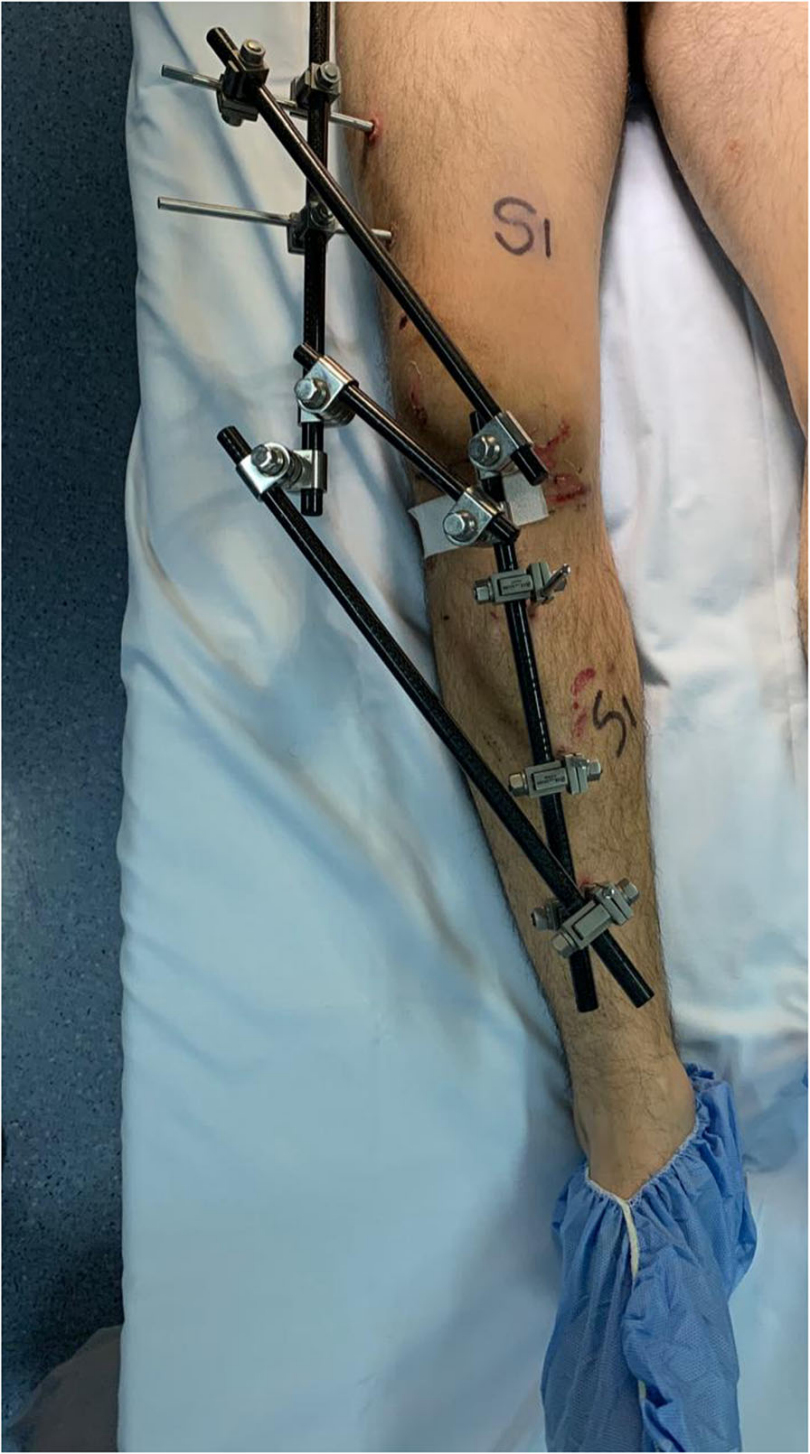
Rigid external fixator

The **Third controversy** refers about the type of ideal pre-operative and post-operative immobilization for those knees with KDIII / IV lesions. In this topic, there is no level I evidence that recommends some type of immobilization over another, but in general it is preferred that in lesions of the four major ligaments with knees that displace after reduced, grossly unstable, and post repair or reconstruction of the ligaments, the hinged external fixator is better than the rigid one, (Fig. 4. Rigid external fixator) because it has the advantage of stabilizing and allowing early mobility, reducing the risk of arthrofibrosis, characteristic of these extensive soft tissue injuries [107]. In addition, some authors such as Lucidi [68], Marcacci [70], Zaffagnini [125], and Angellini [1], recommend it after surgery to protect the ligament reconstruction with good results, especially when posterior cruciate ligament surgery has been performed. Stannard et al. [106] in a prospective randomized study of 77 patients with severe instability found that in patients who had an hinged external fixator placed, ligament reconstruction failed significantly less than those who had a traditional brace. In the authors article opinion, whenever the use of an external fixator is required, whether due to vascular repair, open dislocation or severe instability, the hinged fixator should be preferred because it reduces the risk of arthrofibrosis in these patients, which it has a worse prognosis than residual instability, nevertheless if for any reason was chosen a rigid fixator, it is advisable to remove it between 3 to 4 weeks, recover knee motion and then do the ligament surgery.

**Fig. 5:**
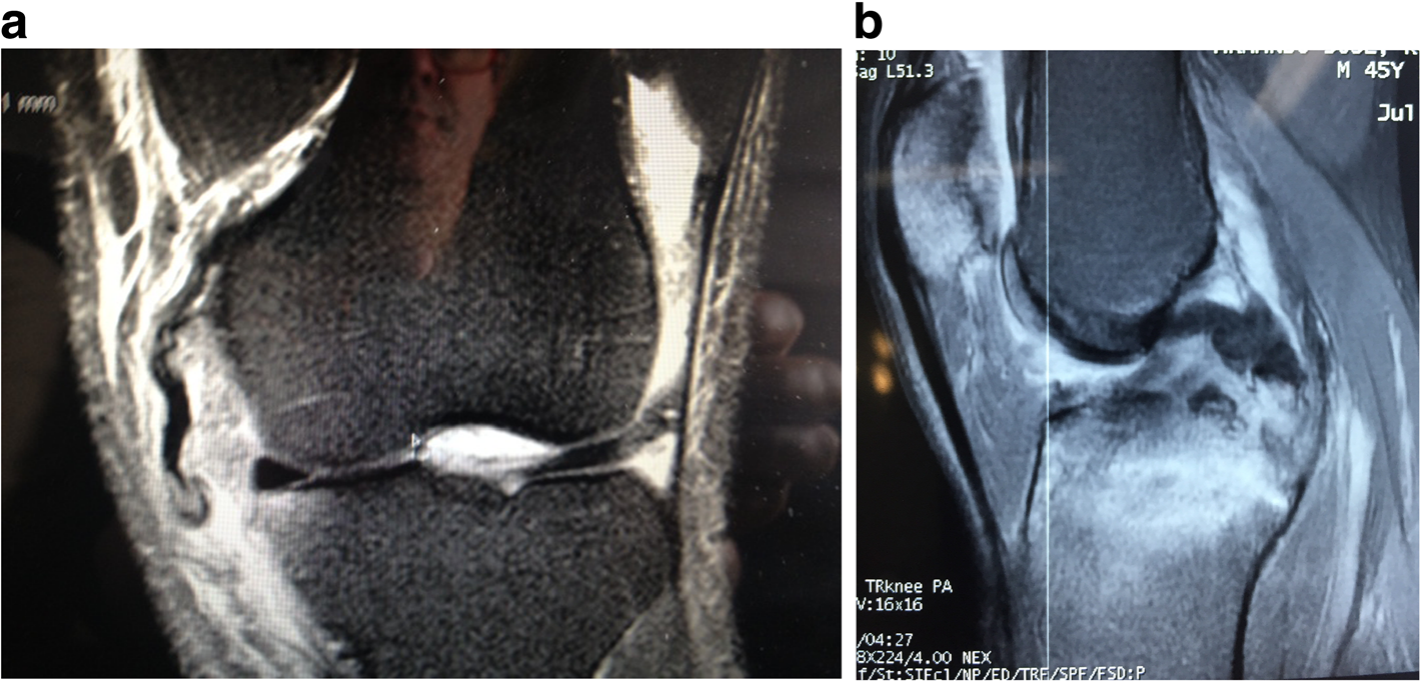
**a** MCL desinsertion. **b** PCL avulsion

**Fig. 6:**
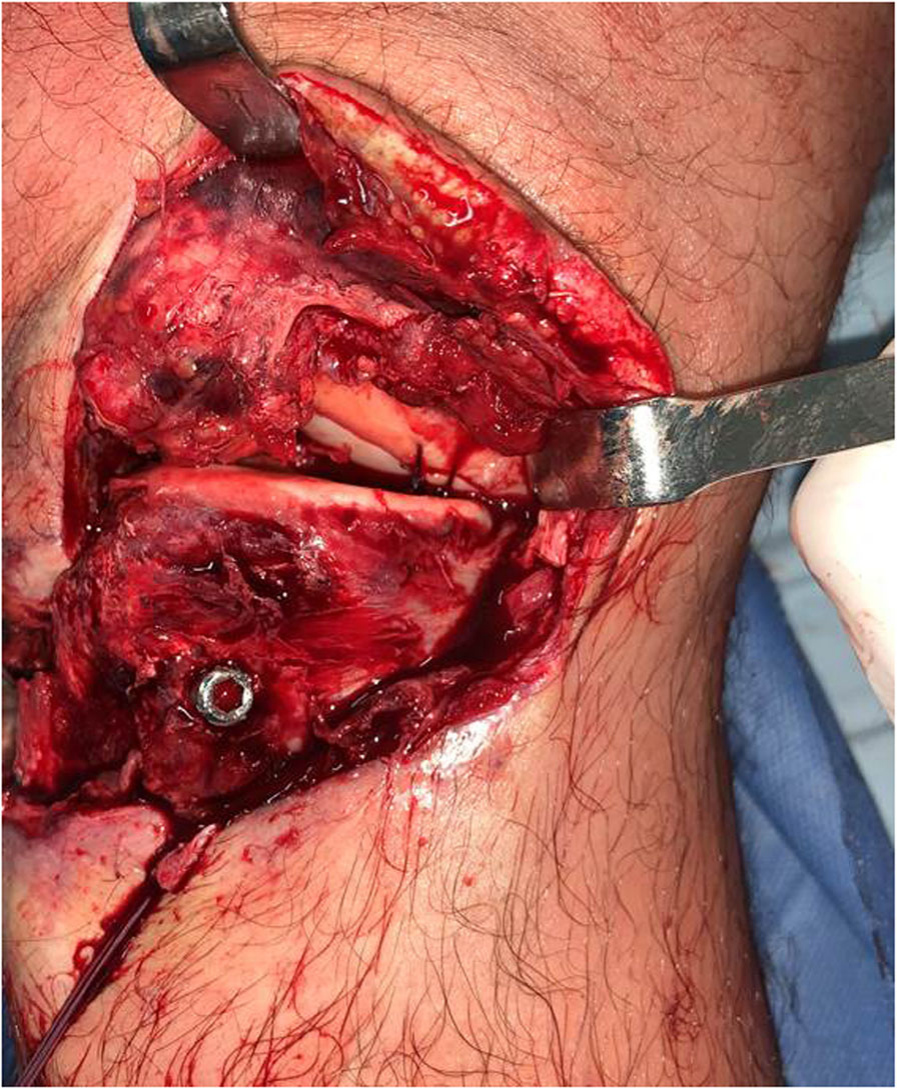
Open Medial Meniscus Repair

**Fig. 7:**
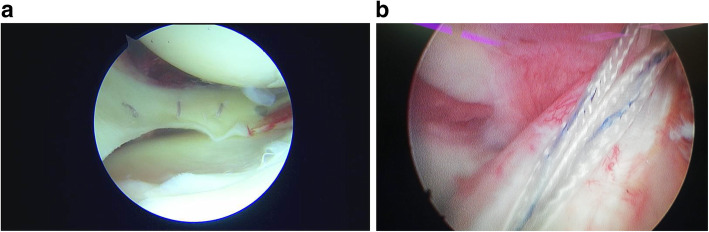
**a** In-out meniscal repair. **b** ACL Repair and Internal Brace

The **Fourth controversy** refers to the ideal time “timing” to operate these patients, and whether it is better to do it in a single or in two stage. Levi et al. [64], in a systematic review and Harner et al. [38] found that early surgery before 3 weeks produces better results in terms of stability, function, return to work and sport than late surgery. Shelbourne et al. [101], Rihn [94], Owens [87] also advocate early surgery before 3 weeks for the difficulty of collateral exploration after this time and the inability to repair them. Vicenti et al. [116] in another systematic review find that it is better to intervene patients before 3 weeks because it produces better results on functional scales. However, other authors prefer surgery in two stages [8, 86, 109]. Mook et al. [79] in a systematic review found similar results in terms of stability and function by performing late or two-stage surgery with similar complications, compared to early surgery. Jiang et al. [47] in another systematic review reported better results in terms of stability in KD/III lesions with two-stage surgery. La Prade et al. [60] in a level IV study in 194 patients reported good results according to the Tegner, Lysholm and Womac scales, with surgery in a single surgical stage performed in acute or chronic phase. The definition in the literature of what it is acute or chronic is confusing and what it is early surgery and late surgery in terms of weeks. In some studies the early one is defined as interventions in the first week, others before the third week, as well occur as in the late surgery that in some cases is define it at 4 weeks and in others at 6 weeks. In general, the authors of this article recommend use the terms of immediate surgery the one that is performed in the first 24 h usually due to vascular repair and open knee dislocation, early surgery that is performed before 3 weeks, and late surgery after 4 to 6 weeks. In this regard, early surgery is recommend it in cases of fixation of bone fracture (KDV), frank lesion of the peroneal nerve, and in the desinsertions / avulsions of the corners and cruciates (Fig. 5a. MCL desinsertion) (Fig. 5b. PCL avulsion). Always the repair procedure should starts with the corners, which include no just ligaments but perypherical meniscus and capsular tissue, to improve knee stability (Fig. 6. Open Medial Meniscus Repair). Once the tissues are closed, we repair cruciates and add some stitchs to the meniscus to strength the repair (Fig. 7a. In-out meniscal repair). On the last 5 years, we’ve repaired all cruciates remnant with high strength sutures and using in some cases augmentation with tapes (Fig. 7b. ACL Repair and Internal Brace). In this situation, it is imperative to continuously check the pressure of the compartments even if they are closed and if the scope is used, to avoid compartment syndromes that will have serious consequences for the patients. The senior author (SA) on more than one occasion has suspended the arthroscopic procedure of repair / reinsertion of the cruciate ligaments due to an unusual increase in the pressure of the compartments and has had to change to the open approach or stage. For irreparable interstitial lesions of the major KDIV ligaments that require reconstructive procedures, it is advisable to perform it as a late surgery after 3 or 4 weeks in a single surgical time to decrease the risk of compartment syndrome and soft tissue problem healing.

**Fig. 8:**
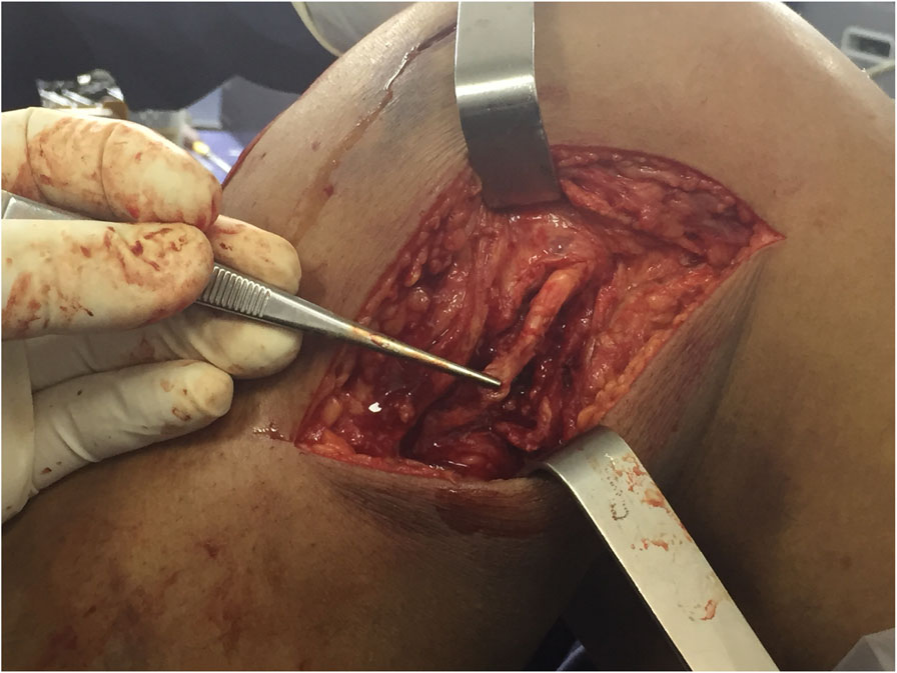
FCL avulsion

**Fig. 9:**
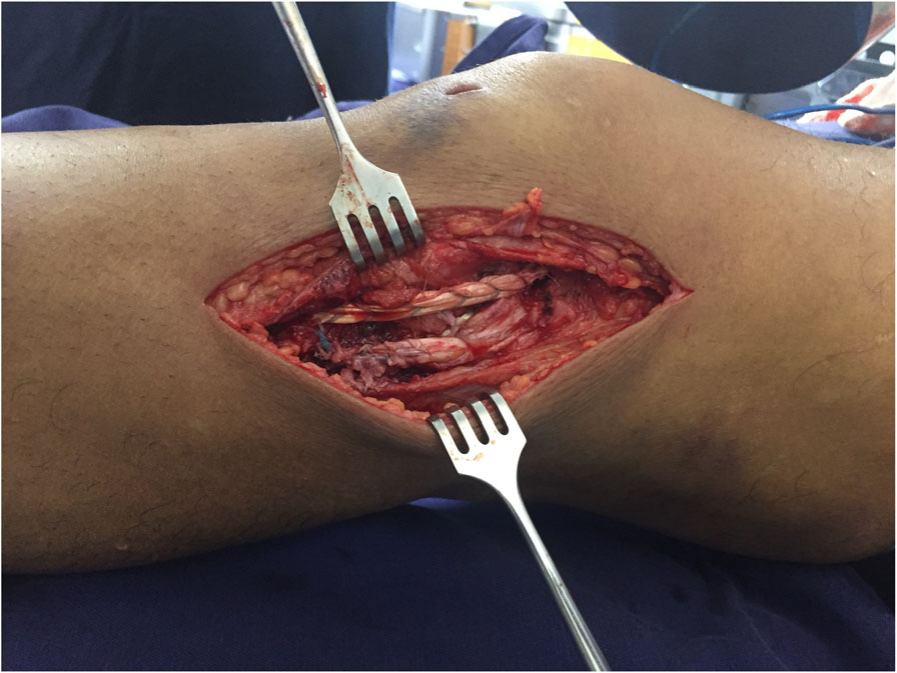
Arciero Technique

**Fig. 10:**
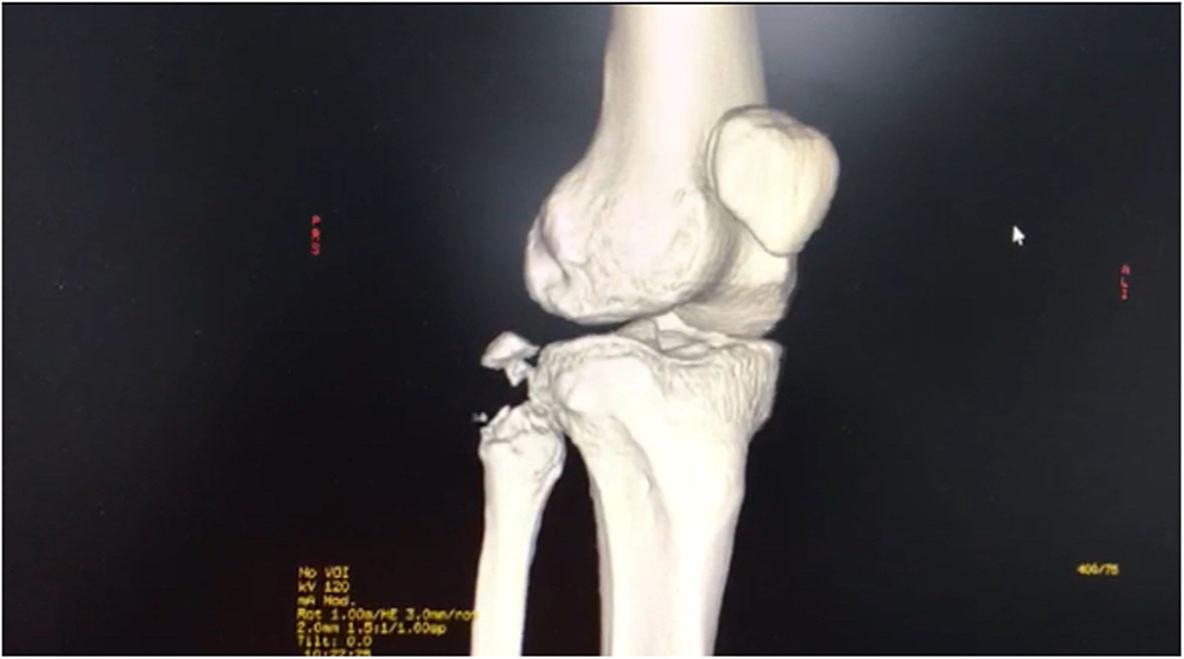
Fibular Fracture

**Fig. 11:**
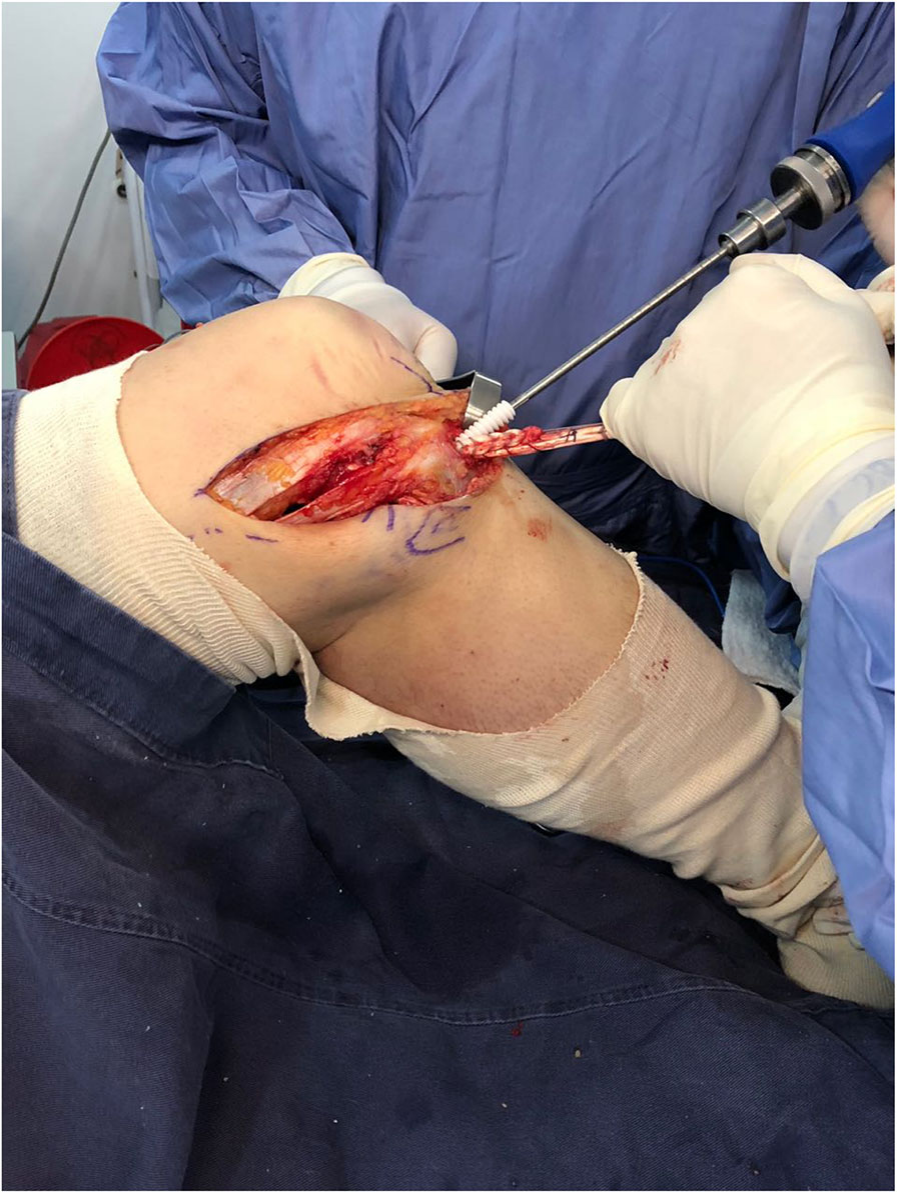
Laprade tibial tunnel

The **Fifth controversy** refers whether it is better to repair than to reconstruct the ligaments and starting for the posterolateral corner, an attempt has been made to generate a consensus [14] with experts regarding the diagnosis, treatment and rehabilitation of these lesions. Different authors such as Stannard et al. [105] in a comparative study between isolated repair versus reconstruction have shown a failure rate greater than 37% versus 9% when repaired, just as Levy et al. in an article already cited found greater failures with repair than with reconstruction. Geeslin et al. [32] in another systematic level IV review, analyze the results of acute repair of the posterolateral corner and conclude that the failure rate is high when cruciate surgery is performed in a second stage. Conversely, Westermann et al. [120] in a multicenter comparative study between repair and reconstruction found no differences in functional scales. For the authors of this article, the results shown in the literature besides to be variables, have low evidence, therefore no conclusions can be given. In early-intervened PLC injuries, which are usually type B and C lesions according to the Bleday and Fanelli classification [9], it is advisible repair / reinsert (Fig. 8. FCL avulsion) and augment with an allograft/auto using preferably the Arciero technique [2, 41, 113] which it is enough to control the varus and external rotation, (Fig. 9. Arciero Technique) without the need to add another tunnel in the tibia [99], which increases the time and trauma of the tissues. In some circumstances there is associate a fibular head fracture that compromises any augmented reconstructive procedure in it (Fig. 10. Fibular Fracture). In late phase or chronic lesions, if a PCL lesion is associated, the authors recommend the Laprade reconstruction technique, because the graft that crosses the lateral tibial tunnel is synergistic with the PCL graft, maximizing the posterior stability of the knee (Fig. 11, Laprade tibial tunnel). In cases of combined ACL and PLC chronic injuries, the Arciero technique produce very good results.

For the postero medial corner there is a consensus that these lesions can heal without surgery and that is why many schools wait 4 to 6 weeks and then only address cruciates ligaments after that. However, De Long [21] in a systematic review concludes that the repair of the posteromedial corner gives excellent results in terms of stability and function. Ferrari et al. [71] prefer in KD/IIIM injuries, to reconstruct all structures in a single surgical time as well as Richter et al. [92] For the senior author, except for distal avulsions or entrapment ligament into the joint that are indicative of surgery, the postero-medial corner lesions heal properly without surgery as long as the limb is not loaded too early. If after 6 weeks the medial side is unstable in extension, he performs ligament capsule plication with excellent results comparable to reconstructive techniques and only in severe post-medial instability with insufficient original tissue, use Lind [66] or Laprade [71] techniques. The possibility of coalescence of tunnels with postero medial reconstructive surgery is high and hence some techniques have been described to avoid it [13, 77, 98]. The medial plication has the advantage of not using grafts, the tunnels are avoided, the original tissue is used and the original insertions are preserved. The senior author in 26 patients who had used this technique due to chronic medial instability, 24 patients had an excellent result according to the IKDC scale. One of the authors of this revision (AJ), use just one femoral tunnel in case of MCL reconstruction to diminish the complications described before and having good results.

**Fig. 12:**
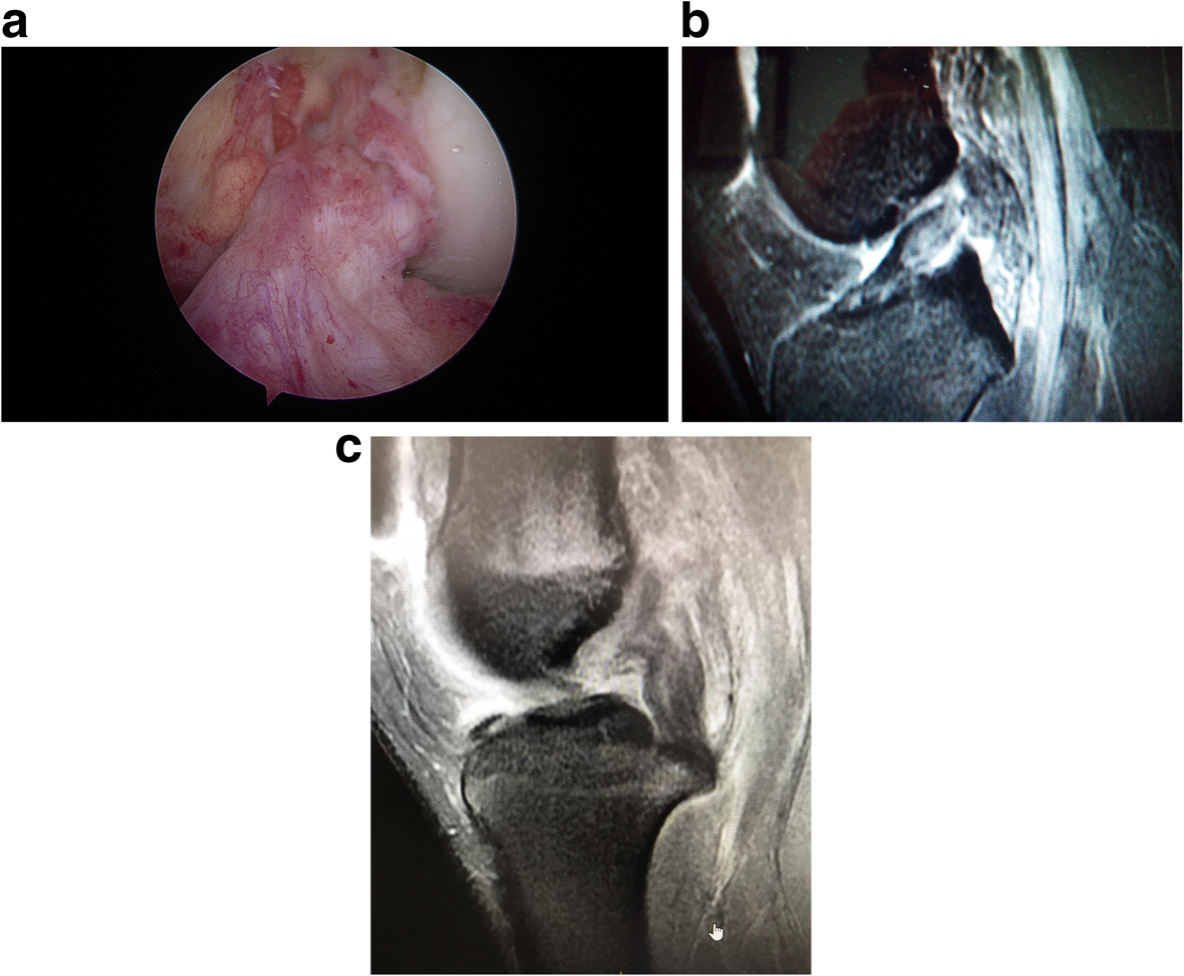
**a** ACL proximal rupture. **b** MRI proximal ACL rupture. **c** Proximal PCL rupture

**Fig. 13:**
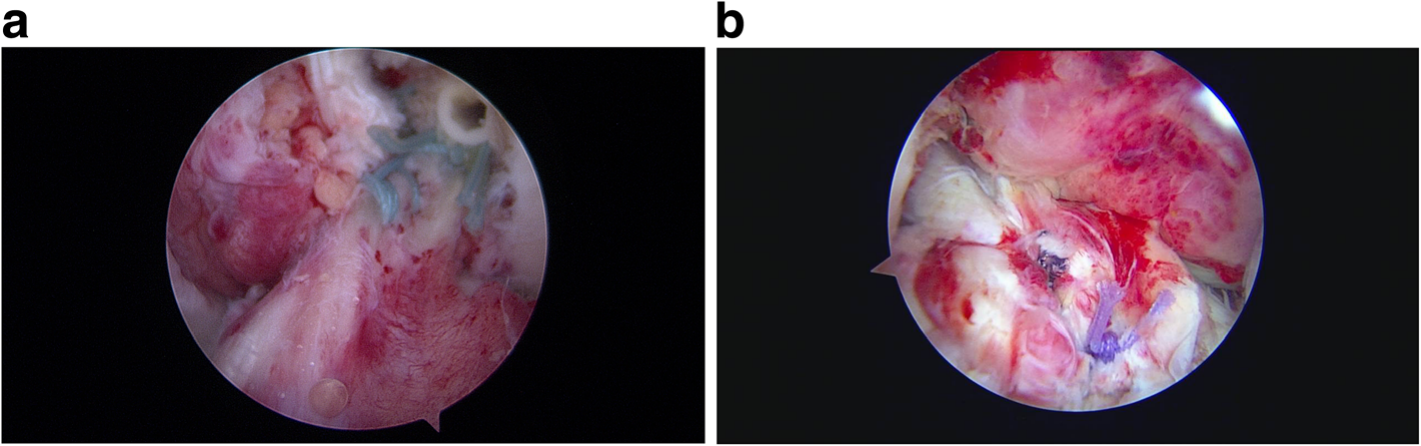
a. Proximal ACL repair. b. Distal ACL repair

**Fig. 14:**
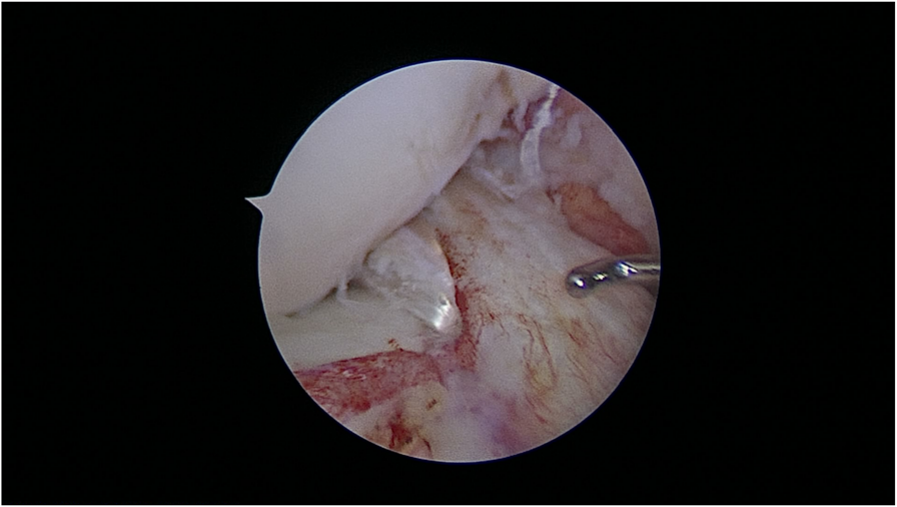
ACL repair and Biological augmentation

**Fig. 15:**
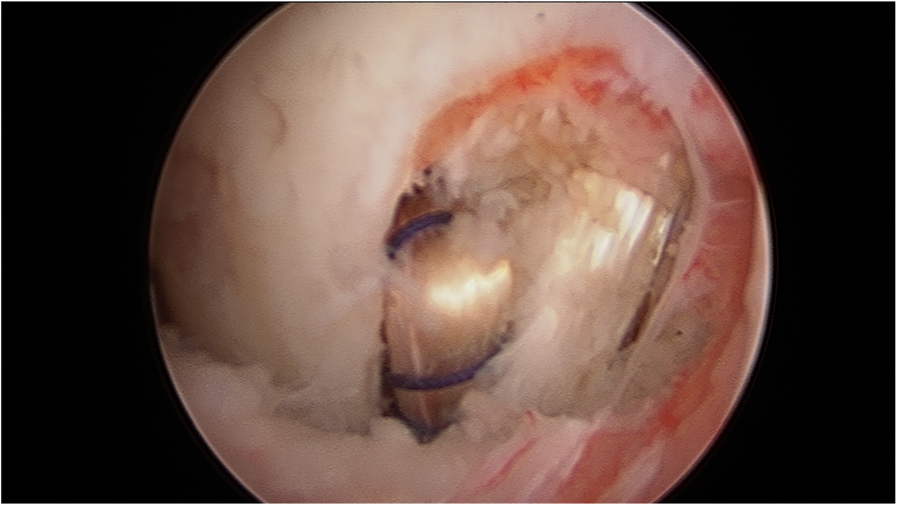
PCL Postero medial Biological Augmentation

**Fig. 16:**
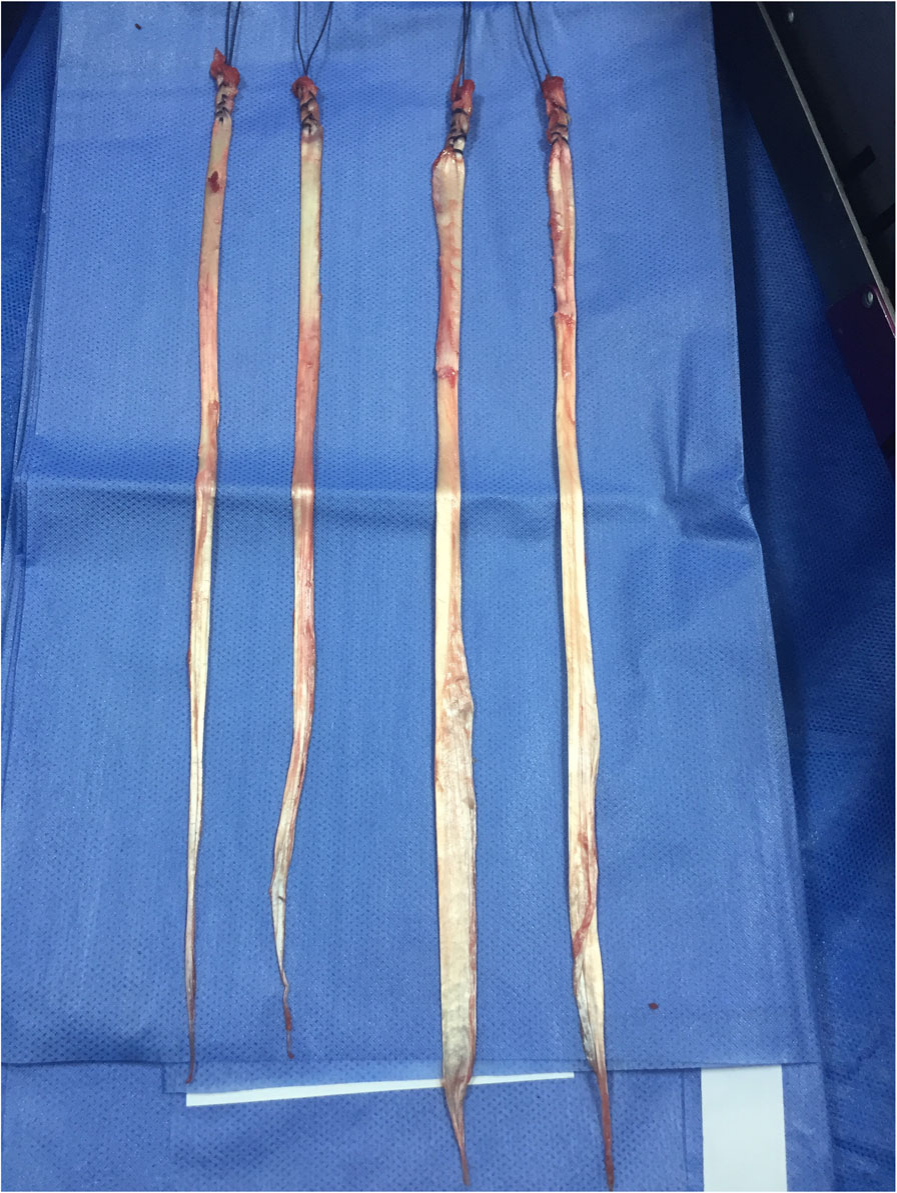
Four Hamstrings

The senior author in KDIV lesions that need be operated early to repair the PLC, the medial side is left alone without any surgical gesture and at the most, the tissue is augmented with ultra-high tension sutures tapes following the principles of the internal brace [45, 75]. There is also controversial if it is better to repair the cruciate ligaments than to reconstructed although different systematic reviews have shown similar results. Heitmann et al. [39], in 69 knee dislocations who had both cruciates repaired and augmented with high tension sutures, obtained 87.5% of good results according to the Lysholm scale. Kohl et al. [55], in 35 patients with MLKI, obtained 82% of good results using for the ACL the Dynamic intraarticular System (DIS, Lygamis™), and repairing the PCL and the corners. On the senior author experience, many of the cruciates injuries in MLKI preserve remnants, (Fig. 12a. ACL proximal rupture), (Fig. 12b,MRI proximal ACL rupture) (Fig. 12c. Proximal PCL rupture) preferably indicating ligament repair / reinsertion over reconstruction (Fig. 13a. Proximal ACL repair) (Fig. 13b. Distal ACL repair), with internal brace augmentation, using high tension sutures with independent fixation [18, 49] or use biological augmentation with autografts (Fig. 14. ACL repair and Biological augmentation) (Fig. 15. PCL Postero medial Biological Augmentation). In the absence of remnants, the article authors prefer use of autografts, taking preferentially the hamstrings of both legs (Fig. 16. Four Hamstrings).

**Fig. 17:**
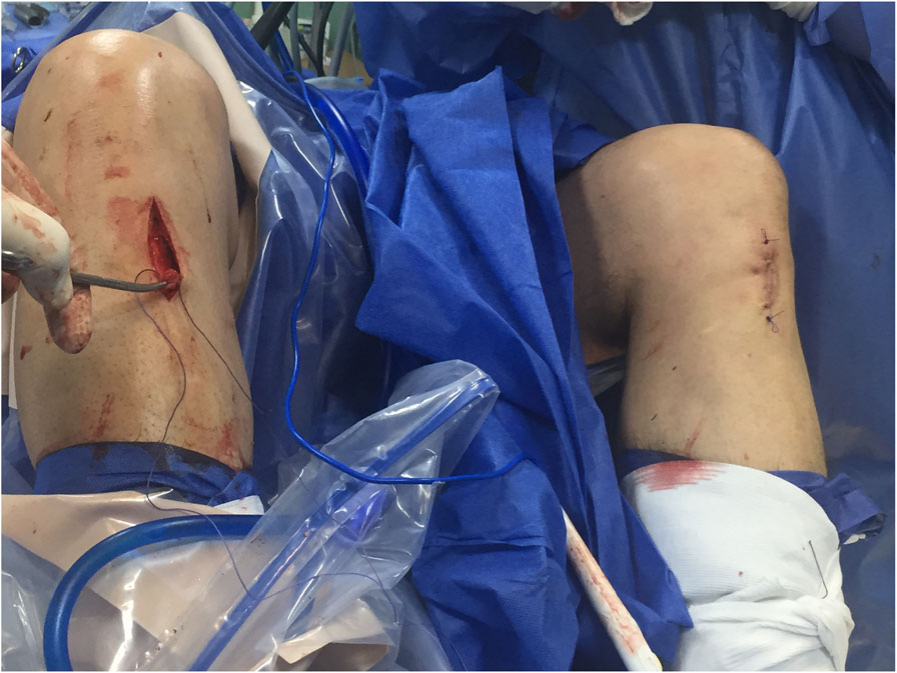
Both leg haversted

The **Sixth controversy** refers to the type of grafts to be used. There is no comparative study that demonstrates any superiority of autografts versus allografts in the treatment of MLKI, but in general the use of the latter is preferred because decrease tissue trauma and surgical time, getting good results regarding to the stability and function [7, 17, 100, 108] however with the use of allografts has been reported greater risk of infection transmission, rejection, and early failure [51, 111]. Franciozi et al. [28] in a case series study, report good results using autos with hamstrings in early MKL surgery. In the experience of the senior author, allografts are a good alternative because of the advantages already stated, but it has been reported high failure in cruciate reconstruction specially in young and active patients. Even in industrialized countries, allografts are not always available, so surgeons we have to take the patient’s tendons, in that sense, the author article recommend the use of the two-leg hamstrings, (Fig. 17. Both leg harvested) minimizing trauma of the affected knee, and decreasing the possibility of failure of the intra-articular surgery reported with the use of allografts, taking into account that most of these patients are young adults, who are going to be exposed to heavy loads in their work and / or sports life. Some authors use synthetic grafts type LARS [91] (Ligament advanced Reinforced System™). Gliatis et al. [48] in a retrospective study of 31 cases report good results using it for PCL in acute cases with the advantage of a smaller surgical trauma and faster return to work activity. Ranger et al., in 111 acute MLKI lesions also used LARS, obtaining 90% of good and excellent results when they reconstructed the ACL and the corners but only 60% of the patients with PCL reconstruction obtained good results. Chiang et al. [15] in a case series study in 33 patients found partial rupture of the LARS in a 10-year follow-up, therefore they do not recommend it.

The **Seventh controversy** refers to the sequence of graft fixation. There is no solid evidence in the literature that supports the best protocol for sequential graft fixation in multi-ligament knee surgery. It has been traditional to first fix the PCL as the central axis of rotation of the knee and main stabilizer [78], however in recent publications made in cadaver studies, it has been seen that tensioning the two cruciates and fixing the ACL first, reproduces better the kinematics of the knee [29, 126] . Kim et al. [53], in a series of case studies in 25 patients with KDII / III / IV, used first tensioning and fixation protocol of the PCL in 14 patients and in 11 both cruciates were tensioned at the same time and fixed the ACL first, obtaining in this last, better posterior stability and functional scales. In the opinion of the senior author, in injuries of the two cruciates, if the PCL is first fixed, without tensioning of ACL graft, there is a risk of fixing the knee not in a neutral position but anterior, generating a tension in PCL graft, which can cause elongation and fail over time. The senior author, currently in KDII/IIIL and KDIV lesions, follows the principles of Kim study tensioning both cruciates, fixing the ACL first, then the PCL, PLC and finally PMC. For the time being, having no randomized prospective comparative clinical studies, the fixation sequence will remain subjective and dependent on the surgeon.

**Fig. 18:**
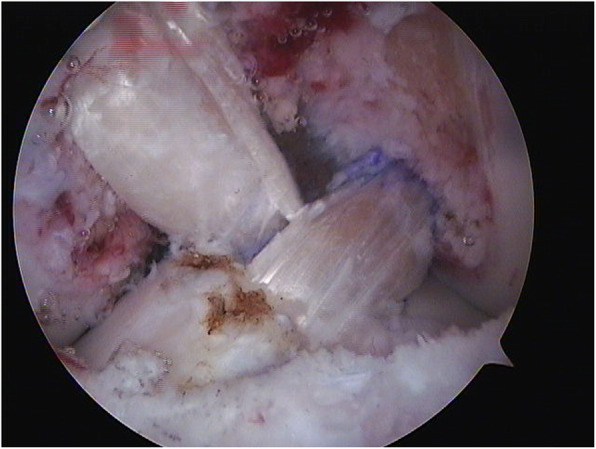
PCL and ACL one bundle reconstruction

The **Eighth controversy** has not been much debated in the literature specifically in the context of multi-ligament surgery of the knee and refers to whether the results in terms of functional scales are better if “anatomical” techniques are made for the reconstruction of the collateral ligaments and for the cruciates, this last with the use of double tunnel. For PLC reconstruction, in cadaver studies conducted by Treme et al. [113], both the Arciero and LaPrade techniques showed similar behavior in terms of varus control and external rotation. In clinical studies the two techniques have also been compared with similar results [114, 115] however other authors such as Kandeel [50] perform non-anatomical biceps tenodesis surgery with results comparable to the techniques described. Little attention has been made about the Postero medial corner but there is a trend to reconstruct the POL and MCL instead of the MCL alone. In PCL reconstruction surgery, the literature referring to the advantages of some reconstructive technique over another is very poor, however in a systematic review in cadaver studies, Lee et al. [62] found that the double bundle gives more posterior stability than a one bundle. Maradei-et al [69] found that the technique of a one bundle with a thick graft is superior to the double bundle. Xu et al. [124] in a prospective comparative study in 59 patients, found no differences between the two techniques. In the authors article opinion, since there is no significant difference between the two techniques and in the multiligament surgery scenario where trauma and surgical time count, recommends the reconstruction of anterolateral band of the PCL with a tick graft, and central-central ACL, reducing trauma and time. (Fig. 18. PCL and ACL one bundle reconstruction). It is noteworthy that the most of the PCL’s injuries that accompany the MLKI are accompanied by a good remnant that can be repaired/reinserted, and augmented with high-tension sutures or grafts.

**Fig. 19:**
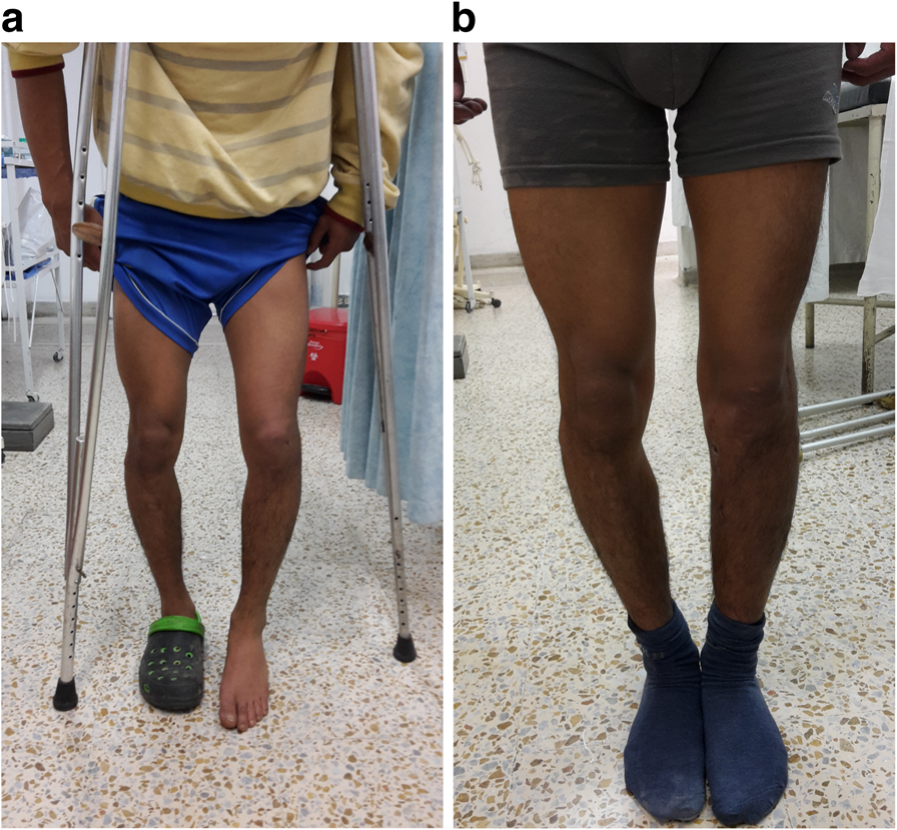
**a**. High Varus deformity. **b** POP valgus osteotomy

The **Ninth controversy** refers to performing coronal or sagittal realignment procedures before reparative or reconstructive ligament surgery. In general, there is a consensus that established chronic multi-directional instabilities, with varus alignments that are exaggerated with gait, such as varus thrust, realignment osteotomy is mandatory to subsequently protect the reconstruction of the corner [6, 12, 81] Noyes et al. [83, 84] found that one of the failures in PLC surgery is not to correct the varus. Arthur et al. [4], in a prospective study in 21 patients with posterolateral instability, found that tibia valguizing osteotomy was effective and 38% of them did not need a second procedure when instability was not severe. Tischer et al. [112] in a systematic review found that the coronal and sagittal angular deformities constitute a failure factor in uni and multiligamentary surgery and that in addition the re-aligning surgery is effective in the treatment of posterolateral instability and in ACL revision surgery. There is no literature support to back up an osteotomy for valgus deformity in PM instability. In what exists controversy is whether it is indicated to do these procedures in acute phase in patients with gross angular deformities especially in varus deformities with injuries of the Posterolateral corner. The literature is extremely poor in this regard and in general surgeons do not indicate it, but the authors article consider that the acute condition does not change the risk factor and failure in posterolateral and posteromedial instability with varus or valgus deformity, therefore, they performs osteotomy as the first surgical procedure in the setting of acute cases with moderate to severe angular deformities (Fig. 19a. High Varus deformity), (Fig. 19b. POP valgus osteotomy). In this scenario, osteotomy is performed after the patient recover full knee motion and in some selected cases, especially in young patients, the osteotomy could be performed at the same time with the ligament reconstruction avoiding a second stage procedure.

The **Tenth and final controversy** refers to rehabilitation, as with surgical treatment, there is little evidence of the ideal rehabilitation program. In general in reparative surgery it is preferred to protect the structures by delaying the beginning of mobility and in reconstructions where rigid implants are used, early motion is allowed [65, 105]. Irrgang et al. [44], recommend that rehabilitation protocols should be individualized taking into account the repaired or reconstructed tissue, and the fixation used. Some authors such as Skendzel [103] and Edson [23] advocate conservative protocols that delay mobility and support, to allow good healing and joint stability. Others like Stannard [105], Noyes [85], and Hubert [43] recommend reconstructive surgery with early motion to avoid complications such as arthrofibrosis which is common in this type of surgery. Mook [79] in a systematic review found that patients operated acutely have more chance of develop arthrofibrosis than late or two-stage surgery and therefore recommends an aggressive rehabilitation program in patients undergoing multiligament repair surgery. There are variable and low scientific evidence studies that deal with the effect of weight bearing in Multiligament surgery, some recommend late support after 6 weeks [22, 26, 46] others immediate partial support and some more aggressive early support without restrictions [44]. Mosquera et al. [80], in a prospective randomized study in 20 patients who had undergone reconstructive corner surgery found that early support affected joint stability in posterolateral corner surgery but not in the posteromedial side. Conversely, LaPrade et al. [61] in a randomized prospective study in 36 patients who had undergone reconstruction of the PLC, found no differences in stability and function at 6 months post-surgery, therefore recommend early support. Finally, although there are reports of patients who have managed to return to their same sport level [57, 104]. Everhart et al. [24] in a systematic review in 26 studies with 524 patients found that the return of high competition sports in multiligament surgery is very low and varies between 22 and 33%. The Authors of this article promote early passive motion starting next day after surgery encouraging knee extension and allowing partial weight bearing after three weeks when corners were addressed. In bicruciate surgery early support is stimulated as soon as the patient tolerates it. Finally in this complex pathology return to contact sports is discouraged to the non-professional athletes. Competitive professional athletes have other motivations that go beyond medical advice (Table 1).

## Conclusions

According to the reviewed literature, most of the articles that refer about the general controversies in the diagnosis and treatment of MLKI lesions are cases series with a low level of clinical evidence. The only topic that there is a currently consensus is that surgical treatment offers better functional results and return to work and sports activity than non-surgical treatment. There is a tendency to intervene early these patients in one stage surgical time, repairing collateral lesions when it allowed and preferably using allografts in ligament reconstructions KDII / III / IV injuries. There is also a trend to use high strength sutures in intra and extra-articular ligament repairs as augmentation, with results similar to reconstructive techniques, reducing the morbidity generated by the latter. Open surgery for collateral lesions remains the gold standard but there is a tendency to use less invasive arthroscopic techniques with similar results with the concept of anatomy reproduction. The importance of coronal and sagittal angular deformities is recognized as a factor of failure in chronic multi-ligament surgery and therefore osteotomies are increasingly indicated before ligament reconstruction, however in the setting of the acute trauma, osteotomy is not accepted for the majority of surgeons. Finally, rehabilitation protocols tend to encourage early mobility and early support with better functional results for patients.

**Table 1:** The 10 recommendations for the Orthopedist dealing with the Treatment of Acute MLKI Injuries

**1**	Always work as a team In the Emergency Room. Two heads think more than one.
**2**	Always, in all cases, rule out a Vascular lesion with accurate tests. Pedal pulse may not be your best friend.
**3**	Always in all cases, rule out a Peroneal Nerve injury with clinical test. The exam of the other leg will help you to diagnose partial injuries.
**4**	Always use an external fixator after a vascular repair. Use hinged if is available. Control Motion is better than not.
**5**	Always accompany yourself in surgery with trained personnel. This is not a 5 min surgery.
**6**	Always reinsert/repair the ligaments avulsions early. The best graft will never be better than the original tissue. Have high strength sutures, tapes, anchors, knotless on hand.
**7**	Always use appropriate measures to reduce surgical trauma in acute surgery. Use allografts instead autografts. Apply anatomical principles to repair /reconstructed ligaments. Remember than in this complex situation, the simple is better.
**8**	Always recover joint motion as soon as possible. Stiffness has a worse prognosis than residual instability.
**9**	Always Correct dynamic or severe angular deformity in chronic cases, before ligaments reconstruction. A varus knee deformity will spoil out your best PLC reconstruction.
**10**	Always remember that these lesions are not comparable in their results with mono-ligamentary surgery. Probably patients never will go back to their same level work and sports. Do not forget to explain them and family.

## Abbreviations

ACL: Anterior cruciate Ligament
CTA: Angio TAC
IKDC: International Knee Documentation Committee
KD: Knee dislocation
LARS: Ligament advanced reinforced system
MLKI: Multi Ligament Knee injuries
PCL: Posterior cruciate Ligament
PLC: Postero Lateral Corner
PMC: Postero medial corner
PM: Postero medial
